# Treating Hearing Loss: From Cochlear Implantation to Gene Therapy

**DOI:** 10.1002/advs.202509960

**Published:** 2025-09-23

**Authors:** Fan‐Gang Zeng, Jieyu Qi, Chen‐Chi Wu, Yilai Shu, Renjie Chai

**Affiliations:** ^1^ Center for Hearing Research Departments of Anatomy and Neurobiology Biomedical Engineering Cognitive Sciences, Otolaryngology‐Head and Neck Surgery University of California Irvine Irvine CA 92697 USA; ^2^ Department of Radiology Zhuhai People's Hospital The Affiliated Hospital of Beijing Institute of Technology Advanced Technology Research Institute School of Life Science Beijing Institute of Technology Beijing 100081 China; ^3^ State Key Laboratory of Hearing and Balance Science Beijing Institute of Technology Beijing 100081 China; ^4^ Advanced Technology Research Institute Beijing Institute of Technology Jinan 250307 China; ^5^ Department of Otolaryngology‐Head and Neck Surgery National Taiwan University College of Medicine Taipei 100233 Taiwan; ^6^ Department of Medical Research National Taiwan University Hospital Hsin‐Chu Branch Hsinchu 302041 Taiwan; ^7^ ENT Institute and Otorhinolaryngology Department of Eye & ENT Hospital Fudan University Shanghai 200031 China; ^8^ Department of Otolaryngology Head and Neck Surgery Zhongda Hospital State Key Laboratory of Digital Medical Engineering Jiangsu Provincial Key Laboratory of Critical Care Medicine School of Life Sciences and Technology School of Medicine Advanced Institute for Life and Health Southeast University Nanjing 210096 China; ^9^ Co‐Innovation Center of Neuroregeneration Nantong University Nantong 226001 China; ^10^ Department of Otolaryngology Head and Neck Surgery Sichuan Provincial People's Hospital University of Electronic Science and Technology of China Chengdu 610072 China; ^11^ Southeast University Shenzhen Research Institute Shenzhen 518063 China

**Keywords:** auditory neuropathy, cochlear implantation, DFNB9, gene therapy, hereditary hearing loss, Otof

## Abstract

Gene therapy has recently restored natural audibility in humans with OTOF‐gene mutations. This biological restoration of hearing is different from cochlear implantation (CI) that produces artificial hearing via electric stimulation of the auditory nerve. In three published trials, 21 DFNB9 patients show 244 grade I/II adverse events (AEs), 2 grade III AEs and no serious AEs. The average gene therapy effect is substantial (52.4‐dB improvement from baseline complete deafness), rapid (0.74‐month time constant), and stable over the initial six months. However, individual outcomes vary from restored normal audibility (≤20 dB HL) to severe hearing loss (≥80 dB HL). Here critical knowledge gaps in gene therapy are identified such as understanding the individual variability, assessing temporal processing, and comparing efficacy with cochlear implantation. It is predicted that gene therapy will include patients with less than complete hearing loss and target other monogenic forms of congenital deafness. Advanced technologies in minimally invasive drug delivery and gene editing will further increase the safety, efficacy and applicability of gene therapy for hearing loss. Cochlear implantation likely remains the standard intervention for severe‐to‐profound hearing loss, but gene therapy will emerge as a viable alternative in treating monogenic forms of deafness.

## Graphical abstract

Cochlear implantation is the primary treatment for deafness, restoring functional hearing in over a million people. Recently, gene therapy has enabled biological hearing restoration in a small number of patients with OTOF‐related mutations. This perspective evaluates both approaches, concluding that cochlear implants will remain the standard for most, while gene therapy may become a viable alternative for certain monogenic deafness.

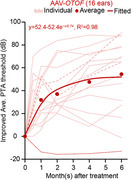

## 1 Introduction

Hearing loss affects 1.5 billion people worldwide, including 403 million with moderate‐to‐complete loss in their better ear that poses significant communication, developmental, and social challenges.^[^ ^1^ ^]^ To address severe‐to‐complete hearing loss, cochlear implantation (CI) has been the gold standard and the only intervention, restoring partial hearing to over one million individuals since 1980s.^[^ ^2^ ^]^ Cochlear implants bypass damaged hair cells to directly stimulate the auditory nerve, producing good performance in speech perception, primarily in quiet listening environments.^[^ ^3^ ^]^ The cochlear implant is particularly effective in treating auditory nephropathy, which typically has preserved cochlear hair cells, but damaged synaptic or neural transmission.^[^ ^4^, ^5^ ^]^ However, electric stimulation spreads broadly in the cochlea, which, coupled with the small number of electrodes, severely limits cochlear implant performance and utility, including poor pitch discrimination, abnormal sound quality, listening difficulty in complex environments, and the need for batteries, electronics, and other cumbersome hardware.^[^ ^6^, ^7^, ^8^ ^]^ While attempts are being made to improve signal processing,^[^ ^9^ ^]^ cochlear implants remain unsatisfactory in nearly 20% of patients.^[^ ^10^ ^]^ Reasons for poor performance in these individuals remain unclear.

These cochlear implant limitations have led to the quest for alternatives that may produce more naturalistic hearing restoration. Initially, hair cell regeneration showed promise but has proven to be challenging in mammalian ears,^[^ ^11^ ^]^ something also evidenced in a recent phase I/IIa clinical trial failure.^[^ ^12^ ^]^ Instead, research focused on repairing synaptic transduction in existing hair cells, like auditory neuropathy, is more feasible and productive, at least in the near term.^[^ ^13^, ^14^, ^15^ ^]^ Indeed, five clinical trials (**Table** **1**) are currently pursuing gene therapy in children with mutations on the *OTOF* gene,^[^ ^16^, ^17^ ^]^ which encodes the protein otoferlin essential for synaptic transmission in inner hair cells.^[^ ^18^ ^]^ The choice for treating the monogenic form of *OTOF‐related* auditory neuropathy is unanimous for the five clinical trials because the *OTOF* gene is primarily expressed in the inner ear, minimizing the off‐target risk. Notably, all five clinical trials have used the relatively mature and safe adeno‐associated virus (AAV)‐mediated delivery and measured hearing improvement in terms of objective auditory brainstem responses and behavioral puretone thresholds.^[^ ^19^, ^20^, ^21^, ^22^, ^23^, ^24^ ^]^ In two published studies, AAV1 and Anc80L65 were used, respectively, in humans after establishing their safety and efficacy in transducing *OTOF* genes into inner hair cells in both mice and non‐human primates (e.g., the vector was predominantly localized to the cochlea with minimal off‐target distribution in the liver and brain; normal behavioral, hematological, biochemical parameters with minimal toxicity and no major organ pathology).^[^ ^19^, ^25^, ^26^, ^27^ ^]^

**Table 1:** Detailed information regarding the five ongoing clinical trials (the leftmost column). The number of planned patients is from the Clinical Trial Registration Site (the rightmost column), while that of treated patients is from either the published papers or the news releases (on or before September 19, 2025).

Sponsor or Institution	Treated/Planned (# of patients)	AAV Serotype	Registration URL
Otovia Therapeutics Inc.	10/25	Anc80L65	https://clinicaltrials.gov/study/NCT05901480?cond = NCT05901480&rank = 1
Eye & ENT Hospital of Fudan University	12/70	AAV1	https://www.chictr.org.cn/showproj.html?proj = 194989
Akouos (Eli Lilly and Company)	8/14	AAV80L65	https://clinicaltrials.gov/study/NCT05821959?cond = NCT05821959&rank = 1
Regeneron Pharmaceuticals	16/30	AAV1	https://clinicaltrials.gov/study/NCT05788536?term = NCT05788536&rank = 1#locations
Sensorion	6/12	AAV8	https://clinicaltrials.gov/study/NCT06370351?cond = NCT06370351&rank = 1

So far 52 patients have received *OTOF* gene therapy, with a total of 151 patients being planned if all five clinical trials are completed (Table 1). While the present result marks a significant milestone in treating genetic forms of deafness, several central questions remain.^[^ ^16^, ^28^ ^]^ First, is gene therapy for hearing loss safe and stable? Second, how effective is gene therapy in terms of improving audibility (hearing threshold, see side box) and functional hearing at both individual and population levels? Third, what exactly gene therapy has repaired in terms of underlying mechanisms? Fourth, what must gene therapy achieve for it to compete or possibly replace CI for patients with monogenic forms of deafness? To address these questions, the present paper summarizes all publicly available data from current human clinical trials. Early evidence suggests that gene therapy is safe and stable at least for 18–24 months, the longest follow‐up so far, improving the average puretone threshold from complete deafness to moderate hearing loss (≈50 dB HL). There is also a great deal of individual variability in the outcome. Moreover, there is limited evidence for restored temporal processing and whether gene therapy outperforms CI in functional perception and communication.^[^ ^29^ ^]^ The large individual variability and the lack of evidence for functional hearing are both likely related to the unknown mechanisms underlying gene therapy. Understanding the mechanisms and quantitative comparison with cochlear implants are critical to expanding gene therapy from treating the rare *OTOF* mutations to more common hereditary deafness or even beyond like age‐, drug‐ and noise‐related hearing loss.

## 2 Gene Therapy is Safe, Rapid, and Stable

The three published single‐arm trials^[^ ^21^, ^22^, ^23^ ^]^ showed that the AAV gene therapy is safe and well tolerated, with 244 grade I/II adverse events (AE), 2 grade III AEs, and no serious AEs. The average number of AEs was 12 per patient from the three studies (246/21); the most common AEs were fever (15%),^[^ ^21^ ^]^ increased lymphocyte counts (14%),^[^ ^22^ ^]^ and decreased neutrophil percentage (10%).^[^ ^23^ ^]^ The absence of severe AEs underscores the safety profile of intracochlear AAV delivery, which has been optimized by integrating microneedles with micropumps to locally administrate the drug through the round window membrane (RWM) for minimal trauma and systemic viral exposure. Although preclinical studies and published single arm trial safety data indicate that local AAV‐*OTOF* delivery exhibits limited toxicity (e.g., hepatotropism but no carcinogenesis),^[^ ^19^, ^21^, ^22^, ^23^, ^30^, ^31^ ^]^ the long‐term (>two years) safety of the AAV gene therapy in humans needs to be monitored for potential chronic inflammation, tissue odd‐targeting and late‐onset toxicity due to microneedle‐related surgical trauma and drug propagation from cochlear aqueduct to cerebrospinal fluid, reaching the brain, liver and other vital organs.^[^ ^32^, ^33^, ^34^ ^]^

**Figure 1:**
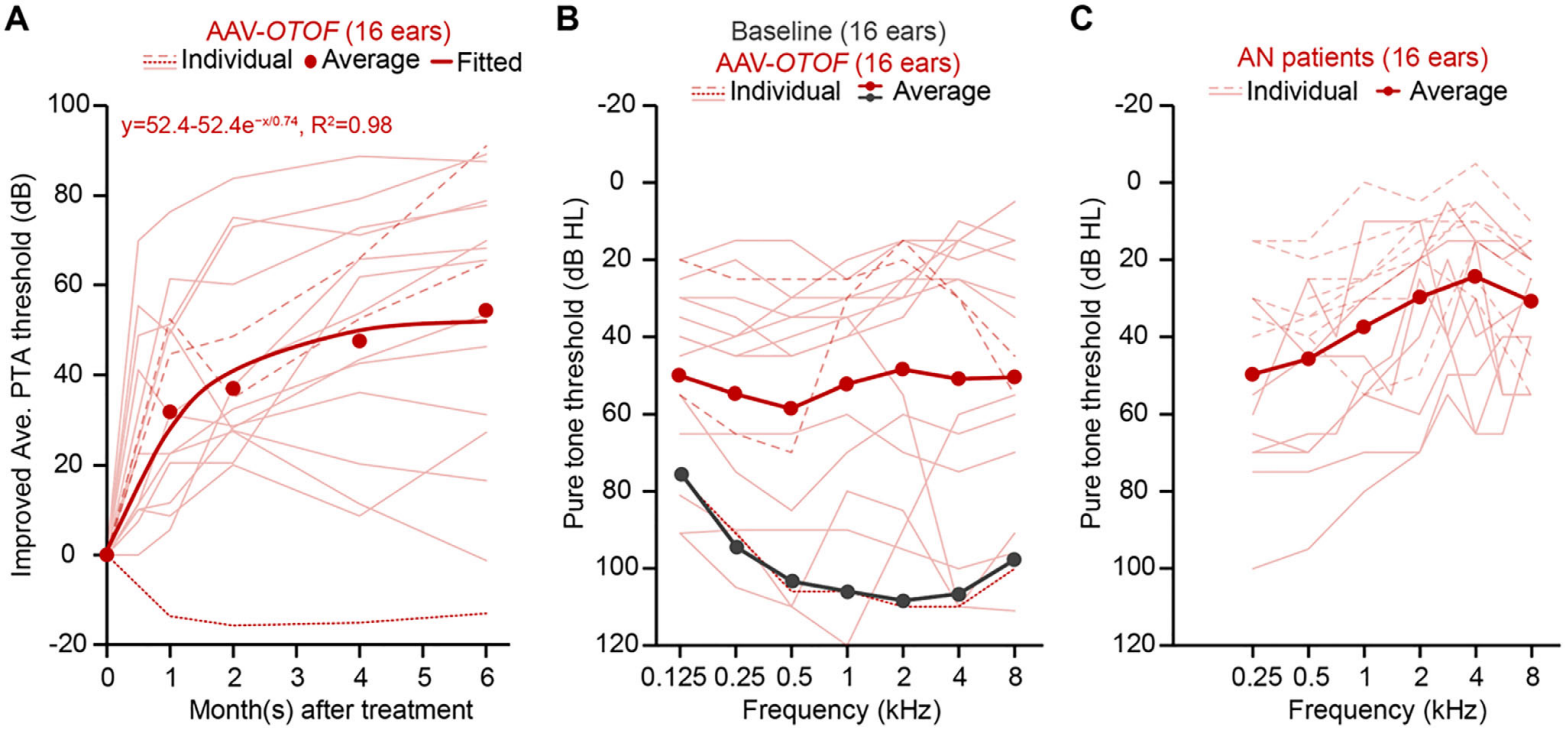
Longitudinal threshold improvement and audiometric profiling across clinical cohorts. A) Time course of gene therapy outcome is shown as the improved thresholds (y‐axis) in terms of the difference in the averaged pure tone threshold between baseline and follow‐ups (x‐axis). Solid red dots indicate the average data. Thin lines represent the individual data (solid lines: 13 ears/10 patients from Qi et al.;^[^ ^23^ ^]^ dashed and dotted lines with the dotted line showing the lone patient without benefiting from gene therapy: 11 ears/8 patients from Lv et al. and Wang et al.^[^ ^21^, ^22^ ^]^). The thick red line shows the fitted exponential growth function of the improved threshold with its equation and the R^2^ value being displayed on top of the panel. B) Audiograms. Pure‐tone thresholds are plotted as functions of frequency for the averaged baseline (dark gray circles and line) and 6‐month post‐treatment (red circles and line). Thin red lines represent individual data at 6 months (solid lines: 13 ears/10 patients from Qi et al.;^[^ ^23^ ^]^ dashed and dotted lines: 3 ears/3 patients with the dotted from Lv et al.^[^ ^21^ ^]^). C) Audiograms in typical auditory neuropathy patients. Solid circles and thick lines represent the average data. Thin red lines represent the individual data (solid lines: 8 ears/10 patients from Zeng et al.^[^ ^35^ ^]^ dashed lines: 8 ears/4 patients in four typical *OTOF* patients from Zeng unpublished data).

Because one must hear sounds first before processing them, restoring audibility is the top priority for any hearing restoration interventions. Thus, we focus on the improved threshold in terms of the difference in hearing levels averaged across audio frequencies before and after gene therapy. **Figure** **1**A summarizes the time course of this hearing improvement (individual data = thin lines; average data = solid circles; fitted exponential function = thick line). Despite great individual variability, which we shall discuss in the next section, the average data show that the gene therapy effect is large (52.4‐dB improvement), rapid (a time constant of 0.74 months), and stable over the initial six months. Pre‐clinical studies suggest the rapid effect is likely a result of high transduction efficiency (60–100%),^[^ ^26^, ^27^ ^]^ fast viral kinetics (1‐3 days from the ear to the blood),^[^ ^26^ ^]^ and early transgene expression (7 days).^[^ ^25^ ^]^ The stable effect is likely a result of sustained *OTOF* transgene expression in post‐mitotic inner hair cells, which lack cell division that may cause expression dilution or silencing.^[^ ^30^ ^]^

## 3 Individual Outcome Variability is High and Hard to Predict

Figure 1B shows audiograms at the six‐month endpoint after gene therapy (individual data = red thin lines; average data = red solid circles connected by red thick line; average pre‐therapy baseline = black solid circles connected by black thick line). On average, gene therapy improves hearing thresholds from profound hearing loss (106 dB HL) to moderate hearing loss (53 dB HL). However, individual outcomes vary over a 90‐dB range from 18 to 108 dB HL, with one patient achieving normal hearing (<20 dB HL) and four patients still having severe hearing loss (90–110 dB HL). Note also that one patient (red dotted line) in the Lv et al. report did not benefit from gene therapy at all. While the average audiogram shows a flat loss, individual phenotypes vary greatly, with most having low‐frequency loss and others having high‐frequency loss, notched or reversed notch loss. The range, variability, and average audiogram are similar to a representative clinical population of patients with varying degrees of auditory neuropathy (Figure 1C). This similarity suggests that gene therapy so far has not restored normal hearing to all patients, but it has clearly alleviated the degree of auditory neuropathy. One extreme scenario is that the *OTOF*‐gene therapy has “cured” auditory neuropathy as evidenced by “restored” auditory brainstem responses, suggesting that the remaining hearing loss is of sensorineural origin due to damaged hair cells. The other extreme scenario is that the gene therapy has not completely repaired the synaptic transmission, which could be reflected by the degree of distortions in waveform morphology of the auditory brainstem response.

To assess gene therapy outcomes, current animal and human studies rely heavily on auditory brainstem responses, which are objective and readily accessible but not without limitations. First, the ABR threshold is the lowest stimulus level that induces a significant increase in the waveform amplitude, which reflects typically the more prominent wave V than wave I. Because wave V reflects neural responses from the brainstem or even the midbrain while wave I reflects cochlear nerve response, restoration of the ABR threshold does not mean full recovery of wave I. Indeed, several animal studies found that the wave I is still distorted or may be half of the normal amplitude in the presence of restored ABR thresholds.^[^ ^13^, ^14^, ^36^, ^37^ ^]^ Similarly, wave I is detected in only 2 of 15 ears in human patients who received the AAV1‐h*OTOF* treatment.^[^ ^38^ ^]^ Second, the ABR and behavioral thresholds are not necessarily associated. This disassociation is a signature of auditory neuropathy^[^ ^39^ ^]^ and has been observed in human patients who received *OTOF*‐gene therapy, especially during the first three months.^[^ ^23^ ^]^ Even if the *OTOF* gene therapy successfully corrects the *OTOF* deficiency, pre‐existing, impaired vesicle release and auditory nerve transmission may persist. Because cochlear biopsies in humans are impossible, new technologies such as synaptic marker imaging are needed to distinguish between gene therapy outcomes and pre‐existing synaptopathy, neuropathy, or other hearing impairments that cannot be repaired by gene therapy. At present, the causes and sites of the remaining hearing loss, if any, in gene therapy remain unclear. As a result, their contributions to the observed outcome variability are unknown.

From a patient's point of view, it is important to know pre‐surgically the outcome of gene therapy, which currently has a 90‐dB range (Figure 1B) from near‐normal hearing to profound loss. The existing literature provides some hints but no definite answers. For example, age may be an important factor, as Qi et al. showed an optimal age range between 5 and 8 years old that produces the most hearing improvement.^[^ ^23^ ^]^ Baseline AAV‐neutralizing titers may be another factor. The only patient who did not benefit from gene therapy, the baseline values were high (1:35, Table 3 in Lv et al. 2024);^[^ ^21^ ^]^ similarly, the adult patient who showed moderate improvement in average thresholds also showed high baseline titer values (1:40, Table 1 in Qi et al. 2025).^[^ ^23^ ^]^ Longitudinal immunological profiling is required to understand the immunomodulatory contribution to the individual variability in treatment efficacy. Furthermore, Qi et al. found that in three patients with simultaneous bilateral injection, two had similar recovery between ears but the other one showed a 26‐dB difference (94 vs 68 dB, patient 10).^[^ ^23^ ^]^ This within‐subjects difference is unlikely due to age, sex, neutralizing antibody or genetic heterogeneity, but might be related to ear‐specific differences in surgery, drug administration, diffusion kinetics and infection efficiency.^[^ ^25^, ^26^, ^27^ ^]^ Finally, no significant correlation was found between outcomes and baseline parameters. At present, the small sample size and the large individual variability, coupled with a lack of understanding of the mechanisms, make predicting individual outcomes a challenge in gene therapy for hearing loss.

## 4 Gene Therapy has not Fully Restored Temporal Processing

While audibility is essential for functional hearing, it alone is insufficient. Patients with auditory neuropathy often complain “I can hear but don't understand you.” This suprathreshold complaint has been attributed to impaired temporal processing, which may be measured by detection of temporal gap and modulation.^[^ ^35^, ^40^ ^]^ Temporal gap detection corresponds to voice onset time or word segmentation, whereas temporal modulation detection to the envelope cues important for speech recognition.^[^ ^41^ ^]^

At present, there is only indirect and limited evidence for improved temporal processing. Because of the young age (1.0‐6.2 years old for six patients in Lv et al.; 1.2–11.0 years old for five in Wang et al.), behavioral measurements were difficult with most data from subjective observation by parents or clinicians. Limited data are available in only four children older than 3.3. years (Participant 1, 3, and 5 in Lv et al. and Patient 1 in Wang et al.). Speech recognition and sound localization were measured and showed significant but highly variable improvement. For example, using the gene therapy ear only, recognition of mono‐, di‐syllabic words and sentences in quiet was 0% at baseline in all four children because they were deaf; at 26 weeks, the recognition score was improved to 74.0%, 88.6%, and 73.6% in Participant 3^21^, 54.0,%, 62.9% and 23.6% in Participant 5^21^, but had surprisingly minimal or no improvement (2.0%, 1.4%, and 0.0%) in the 11‐year‐old, bilaterally‐treated Patient 1.^[^ ^22^ ^]^ At 26 weeks, speech recognition in steady‐state noise was also measured in two children with good performance in quiet; Speech reception threshold, or the signal‐to‐noise ratio at which 50%‐correct is achieved, was ‐2.0, 0.3, and 8.9 dB for monosyllabic words, disyllabic words, and sentences in Participant 1 and 6.4, 9.7, and 29·0 dB respectively in Participant 3.^[^ ^21^ ^]^ In the 11‐years old bilaterally‐treated Patient1, sound localization was measured to have a bilateral root mean square error of 92.8°± 1.1° at baseline and 40.0°± 1.7° at 26 weeks.^[^ ^22^ ^]^ The speech in noise recognition and sound localization performance, while significantly improved from the baseline, was similar to cochlear implant user^[^ ^29^ ^]^ but still far from what would be expected from normal‐hearing controls (e.g., 2‐dB speech reception threshold for sentence recognition in noise and 6.0° error for sound localization).^[^ ^42^, ^43^ ^]^

As mentioned above, it is possible that gene therapy only partially repairs the synaptic transmission, which can support detection of slowly‐varying temporal envelope cues but not fine‐structure temporal ones.^[^ ^40^, ^41^ ^]^ This possibility is supported by a recent report in DFNB9 patients showing a progressively higher incidence of auditory brainstem response waves from the most peripheral component reflecting the auditory nerve activity or wave I (2/15 ears) to more central activities like wave III (9/15) and V (15/15).^[^ ^38^ ^]^ This central possibility is partially supported by an imaging and electroencephalogram study, showing that cortical activities were similar to the baseline at 4–6 weeks but increased at13 and 26 weeks after gene therapy.^[^ ^24^ ^]^ Future studies need to differentiate between the peripheral and central mechanisms underlying temporal processing. Because hearing thresholds and speech performance are not necessarily correlated in patients with auditory neuropathy,^[^ ^35^ ^]^ it is equally, if not more important to measure psychophysical, electrophysiological and other functionally meaningful performance to assess how much and how fast gene therapy restores temporal processing. To probe underlying mechanisms, animal models need to be developed to measure temporal processing such that biopsies may be performed at different time points following gene therapy or experiments that are difficult to justify in humans may be done in animals, e.g., cochlear implantation in one ear and gene therapy in the other ear simultaneously (see next section).

## 5 Cochlear Implantation versus Gene Therapy

Unlike CI, which was considered “bad medicine” initially,^[^ ^3^ ^]^ gene therapy was born with a silver spoon in the mouth and widely considered as a promising breakthrough in treating hearing loss. Like CI, gene therapy initially focused on restoring audibility or hearing thresholds,^[^ ^19^, ^21^, ^22^, ^23^ ^]^ but may follow a similar path to restore functional hearing in monogenic forms of deafness, from speech recognition in quiet and noise to spatial hearing and even music appreciation.

A recent study compared performance between 11 gene‐therapy patients and 61 CI patients who were matched in age and presurgical measures such duration of deafness, hearing thresholds, and speech abilities.^[^ ^29^ ^]^ Overall, gene‐therapy patients showed comparable auditory and speech perception to CI patients at 12 months after treatment, except that gene‐therapy patients appeared to learn the tasks quicker and marginally outperformed the CI patients in 3 out of 46 tasks (i.e., ambient sound detection, *p* = 0.02; disyllable recognition in noise, *p* = 0.03; singing tune accuracy, *p* = 0.04; See eTable 8A). The present limited evidence, along with the known large individual variability in CI performance,^[^ ^44^, ^45^, ^46^, ^47^, ^48^, ^49^ ^]^ makes it difficult to judge which treatment is superior. More consistent matches in patient baseline characteristics, post‐operative follow‐up and learning environments in a larger sample size are needed to answer this question. The ideal comparison would compare the performance between the left and right ear of the same patient with one ear receiving CI while another receiving gene therapy simultaneously. No such data are available at present.

Nevertheless, based on comprehensive understanding of auditory neuropathy and CI as well as preliminary data in gene therapy, we make the following predictions. First, gene therapy may restore sound intensity perception more naturally than CI because healthy outer hair cells in gene therapy patients would produce normal cochlear compression that is absent in cochlear implant patients.^[^ ^50^ ^]^ Second, gene therapy may only partially restore synaptic transmission, leading to impaired temporal processing, whereas electric stimulation produces strong neural synchronization, resulting in better than normal temporal modulation detection.^[^ ^51^ ^]^ Third, gene therapy should produce better pitch discrimination than CI at high frequencies but not necessarily at low frequencies. The reason for this prediction is that high‐frequency pitch perception relies on the place cue, which should have much narrower spread of excitation in gene therapy than CI, whereas low‐frequency pitch perception relies on the temporal cue, which may still be impaired even after gene therapy.^[^ ^52^ ^]^ Because speech contains redundant cues in either the frequency or the time domain, both gene therapy and CI may produce good speech recognition in quiet, but which one is better for speech recognition in noise will depend on not only the degree of restored temporal processing but also the tradeoff between temporal and spectral processing.^[^ ^5^, ^53^ ^]^

Hearing devices and gene therapy are not necessarily mutually exclusive. It is possible that they may co‐exist or even be integrated to improve the overall patient performance and satisfaction. After all, gene therapy cannot repair non‐genetically related hearing loss, as evident by the wide range of mild‐to‐profound post‐treatment hearing loss. Hearing aids are one possible solution to addressing the remaining hearing loss, especially in those patients who have mild‐to‐moderate hearing loss. Cochlear implants are the other solution for those patients with severe‐to‐profound hearing loss. A third possible solution is similar to electro‐acoustic simulation and combined acoustic and electric or hybrid hearing, which has been used to treat patients with residual acoustic hearing in one or two ears.^[^ ^54^, ^55^ ^]^ Timing and the degree of residual hearing are critical to this approach: Gene therapy should be applied first to restore as much impairment as possible, with proper hearing devices being used to treat residual loss if any.

Finally, there has been well‐established evidence for cochlear implantation in quality‐of‐life improvements and cost‐effectiveness.^[^ ^56^, ^57^ ^]^ The global average unit price for cochlear implantation has been stable at US$40000‐50,000^8^ but is 10 times lower in China presently.^[^ ^58^ ^]^ In contrast, there lacks robust evidence for the cost‐effectiveness of gene therapy because the price has not been determined but likely high at least initially due to high R&D cost and low number of available patients. As a reference, the gene‐therapy price ranges from $0.4m for treating inherited retinal diseases per eye to $2m for spinal muscular atrophy per patient.^[^ ^59^ ^]^

## 6 Where May Gene Therapy Go From Here?

### 6.1 Expanding Genetic Targets

In the next 5–10 years, cochlear implants are likely to remain the primary intervention for severe‐to‐profound hearing loss because of their established efficacy, broad candidacy, and widespread availability. In the long run, gene therapy will complement but is unlikely to replace CI. One reason for this prediction is that *OTOF* mutations are a rare disease, affecting only 1–3% of individuals with congenital hearing loss.^[^ ^60^, ^61^, ^62^, ^63^, ^64^, ^65^, ^66^, ^67^, ^68^, ^69^ ^]^ Another reason is that as long as hair cells cannot be regenerated, current gene therapy technology is not suitable to most hearing‐impaired individuals who likely have damaged or absent hair cells due to genetic causes, presbycusis, noise exposure, and ototoxic drugs. Like CI, gene therapy will progress to relax candidacy criteria in DFNB9 patients from complete loss to less degrees of hearing loss, resulting in improved performance or even totally restored hearing. More excitingly, the initial success of *OTOF* gene therapy allows research to target other deafness genes. While similar vector engineering, delivery protocols, and safety assessment may be used, expanding to other genes faces significant challenges.

To date, there have been more than 35 preclinical proof‐of‐concept studies involving more than 20 genes using either gene replacement or gene editing in deaf mice.^[^ ^70^ ^]^ Most deafness gene therapies target hair cells, benefiting from vectors that have been optimized for specific transduction of hair cells. In contrast, much less has been to develop vectors specific to other target cell types, limiting the potential for deafness gene therapy. **Table** **2** summarizes these candidate genes and their prevalence, gene size, and cellular expression.

**Table 2:** Candidate genes based on prevalence, inheritance, expression sites, and gene size. Accession information, OMIMID, and key references are also provided (ChatGPT was used to generate a draft version of this table, which has been verified item‐by‐item manually by Professor Qi).

Gene (Locus)	Inheritance	Estimated Contribution^a)^	Main Expression Site(s)	Gene Size (approx.)	Gene ID	OMIM ID
GJB2 (DFNB1A)	Autosomal Recessive	≈8‐22% of HL ≈16‐25% of HHL^[^ ^64^, ^71^, ^72^ ^]^ ≈11‐57% of NSHL^[^ ^73^ ^]^	Cochlear supporting cells^[^ ^74^, ^75^ ^]^	≈5.5 kb (226 aa)	2706	121 011
STRC (DFNB16)	Autosomal Recessive	≈1.7‐16.1% of HL^[^ ^76^ ^]^ 6% of ARNSHL^[^ ^73^ ^]^	Outer hair cell stereocilia^[^ ^77^ ^]^	≈19 kb (1775 aa)	161 497	606 440
SLC26A4 (Pendred, DFNB4)	Autosomal Recessive	5‐12% of HHL^[^ ^64^, ^72^ ^]^ 3–20% of ARNSHL^[^ ^72^, ^73^, ^78^ ^]^	Spiral ligament, stria vascularis^[^ ^79^ ^]^	≈57 kb (780 aa)	5172	605 646
MYO15A (DFNB3)	Autosomal Recessive	≈5% of HHL 6.2% of ARNSHL^[^ ^64^, ^72^ ^]^	Hair cell stereocilia tips^[^ ^80^ ^]^	≈71 kb (3530 aa)	51 168	602 666
TMPRSS3 (DFNB8/10)	Autosomal Recessive	4% of HHL^[^ ^64^ ^]^ 1–2.5% of ARNSHL^[^ ^81^, ^82^ ^]^	Inner hair cell presynaptic terminals^[^ ^83^, ^84^ ^]^	≈24 kb (454 aa)	64 699	605. 511
MYO7A (Usher 1B, DFNB2)	Autosomal Recessive	≈1‐5% of HHL^[^ ^64^, ^72^ ^]^	Hair cell stereocilia, photoreceptors^[^ ^85^ ^]^	≈87 kb (2215 aa)	4647	276 903
OTOF (DFNB9)	Autosomal Recessive	≈1‐3% of HHL^[^ ^60^, ^61^, ^62^, ^63^, ^64^, ^65^, ^66^, ^67^, ^68^, ^69^ ^]^	Inner hair cell presynaptic terminals^[^ ^18^, ^86^ ^]^	≈102 kb (1997 aa)	9381	603 681
TECTA (DFNA8/12, DFNB21)	Autosomal Dominant/Recessive	≈1‐3% of HHL^[^ ^64^, ^72^ ^]^ 18% of ADNSHL^[^ ^73^ ^]^	Tectorial membrane^[^ ^87^ ^]^	≈90 kb (2155 aa)	7007	602 574
USH2A (Usher 2A)	Autosomal Recessive	≈3% of HHL^[^ ^64^, ^72^ ^]^	Hair cell synapses, retina^[^ ^88^ ^]^	≈800 kb (5202 aa; very large)	7399	608 400
CDH23 (Usher 1D, DFNB12)	Autosomal Recessive	1.6% of HL ≈2‐3% of HHL^[^ ^72^, ^89^ ^]^	Hair cell stereocilia tip links^[^ ^90^ ^]^	≈419 kb (3354 aa)	64 072	605 516
KCNQ4 (DFNA2A)	Autosomal Dominant	<1% of HL^[^ ^72^ ^]^ 2.5% of ADNSHL^[^ ^73^ ^]^	Outer hair cells^[^ ^91^ ^]^	≈57 kb (695 aa)	9132	603 537
TMC1 (DFNA36, DFNB7/11)	Autosomal Dominant/Recessive	3.7% of HHL^[^ ^72^ ^]^ 0.17% of NSHL^[^ ^76^ ^]^	Hair cell stereocilia tip links^[^ ^92^ ^]^	≈317 kb (760 aa)	117 531	606 706
LOXHD1 (DFNB77)	Autosomal Recessive	4% of HHL^[^ ^64^ ^]^ <1% of NSHL^[^ ^93^ ^]^ <1% of ARNSHL^[^ ^94^ ^]^	Hair cell stereocilia tip links^[^ ^95^, ^96^ ^]^	≈180 kb (2067 aa)	125 336	613 072
MYO6 (DFNA22)	Autosomal Dominant	<1% of ADNSHL^[^ ^72^ ^]^ 21% of ADNSHL^[^ ^73^ ^]^	Hair cell stereocilia, Inner hair cell^[^ ^97^, ^98^ ^]^	≈170 kb (1294 aa)	4646	600 970
PCDH15 (USH1F, DFNB23)	Autosomal Recessive	<1% of HL^[^ ^72^ ^]^	Hair cell stereocilia tip links^[^ ^99^ ^]^	≈1825 kb (1955 aa)	65 217	605 514

^a)^ The estimated contribution of each gene varies with regions and ethnicity.

HL: hearing loss; HHL: hereditary hearing loss; NSHL: non‐syndromic sensorineural hearing loss; ARNSHL: autosomal recessive non‐syndromic sensorineural hearing loss; ADNSHL: autosomal dominant non‐syndromic sensorineural hearing loss.

*GJB2* is a priority candidate gene because it is responsible for up to 30% of congenital hearing loss.^[^ ^64^, ^100^ ^]^ However, GJB2's broad expression in the cochlea and its human heterogeneity make it a challenging initial target. Unlike *OTOF* recessive loss‐of‐function mutations, *GJB2* variants, either dominant or recessive,^[^ ^101^, ^102^ ^]^ require a different therapeutic strategy such as allele‐specific silencing or editing. For example, DFNB1, a main form of *GJB2* related deafness,^[^ ^103^, ^104^ ^]^ has ubiquitous expression of *GJB2* across cochlear supporting cells and lateral wall cells.^[^ ^102^, ^105^ ^]^ This ubiquitous expression, coupled with the ectopic expression‐induced ototoxicity in hair cells,^[^ ^106^ ^]^ requires therapeutic AAV vectors with both broad tropism and stringent *GJB2* ^+^ cell‐specific targeting. Different from pure synaptic dysfunction in *OTOF*‐mediated hearing loss,^[^ ^18^ ^]^ *GJB2*‐mediated hearing loss has pronounced phenotypic heterogeneity.^[^ ^107^ ^]^ Because homozygous *GJB2* mutations are embryonically lethal in mice, it is challenging to develop proper *GJB2*‐related deaf mouse models that exhibit both phenotypic and genotypic similarities to humans.^[^ ^108^ ^]^ Current preclinical studies on *GJB2* replacement therapy targeting DFNB1 have primarily shown efficacy in *GJB2* conditional knockout mice and safety in non‐human primates,^[^ ^109^ ^]^ but failed to show that these animal models accurately recapitulate cochlear pathological changes caused by *GJB2* deficiency in humans. Even if heterozygous *GJB2*‐mutant mice may be generated by semi‐cloning technology to avoid placental defects,^[^ ^110^ ^]^ their potential clinical utility is limited by the cumbersome acquisition procedure and high cost.

Carlson et al. recently evaluated potential candidate genes that may be prioritized for gene therapy in humans based on critical criteria like gene size, timing of cochlear degradation, cell types of primary expression, availability of mouse models and efficacy of AAV delivery in those mice, and human hearing loss severity, onset, and prevalence.^[^ ^111^ ^]^ They identified three genes, *TMPRSS3*,^[^ ^112^ ^]^ *PCDH15*,^[^ ^113^ ^]^ and *TMC1*,^[^ ^114^, ^115^, ^116^ ^]^ that satisfy all criteria from a list of 93 non‐syndromic hearing loss genes. Two other genes, *LOXHD1* and *MYO6*, had not yet had gene replacements attempts in mice but were considered promising candidates because all other conditions are met.

We also consider several other genes like *SLC17A8*,^[^ ^117^ ^]^ *STRC*,^[^ ^118^ ^]^ *Myo15A*,^[^ ^80^, ^119^ ^]^ and *CDH23*,^[^ ^90^, ^120^ ^]^ as potential candidates for gene therapy as they exhibit restricted expression in cochlear cell types without broad morphological defects due to non‐developmental mechanisms. *SLC26A4*, which encodes the ion exchanger pendrin, is of interest because its mutations represent the second leading cause of hereditary hearing loss.^[^ ^121^ ^]^ *SLC26A4* has been delivered via rAAV2/1 with a CMV promoter to the endolymphatic sac of deaf mice to restore partial hearing.^[^ ^122^ ^]^ Dual‐ or multi‐AAV vectors approach provided an effective way to deliver the large genes, like *OTOF* ^[^ ^26^, ^27^ ^]^ and *STRC*,^[^ ^118^ ^]^ which successfully improved deaf mice hearing. *Myo15A* and *CDH23* would require a triple‐vector approach to deliver their full coding sequences. The triple‐vector delivery, with each recombinant AAV having <4.8 kb transgene capacity,^[^ ^123^ ^]^ remains a technological challenge.^[^ ^124^ ^]^

### 6.2 Delivery Innovation

At present, 4 out of 5 global *OTOF* gene therapy clinical trials have utilized AAV1 or Anc80L65 (Table 1) for their efficient infection of inner hair cells. In the published studies, two patients showed low positive anti‐AAV neutralizing antibody responses. It is difficult to infer whether and how pre‐existing neutralizing antibodies affect treatment efficacy, as those two patients exhibited different outcomes: One showed no hearing recovery, while the other improved to a moderate hearing loss level. Longitudinal immunological profiling is required to clarify the role of immunomodulation in individual variability of treatment efficacy. AAV new serotypes could be still optimized.

We expect advances in developing safer and more efficient drug delivery. Because AAV particles (20 nm in diameter) cannot penetrate the round window membrane, microneedle (≈200 µm) delivery via RWM penetration represents the only clinically validated method in actual human patients at present.^[^ ^19^, ^21^, ^22^ ^]^ A future approach is less invasively CSF‐mediated AAV delivery, which has been used in eighteen clinical trials for AAV therapy of neurological disorders.^[^ ^125^ ^]^ Not only has cochlear transduction via CSF been applied successfully in adult murine^[^ ^32^ ^]^ and non‐human primates,^[^ ^33^ ^]^ but the connection the cochlear aqueduct to CSF connection was also shown in humans.^[^ ^34^ ^]^ However, it is unclear whether and how interspecies differences in anatomy and physiology will affect the safety and efficiency of CSF‐drug delivery to the human inner ear.^[^ ^126^, ^127^, ^128^, ^129^ ^]^ For example, large AAV volumes are required for the CSF than RWM injection (10 vs 1–2 µL in mice, and 2–4 mL versus 30–40 µL in non‐human primates), inducing potentially stronger immune responses and hepatotoxicity.^[^ ^130^, ^131^ ^]^ Because the CSF circulates throughout the ventricular system and subarachnoid space, delivered AAV may infect cells in non‐target brain regions, triggering unintended gene expression, neurotoxicity, or even behavioral changes.^[^ ^32^, ^132^, ^133^, ^134^ ^]^ Finally, CSF injection needs to take both interspecies and intraspecies differences into account. The cochlear aqueduct is narrower and longer in primates than mice, impeding CSF flow to the inner ear.^[^ ^129^, ^135^, ^136^, ^137^ ^]^ Human cochlear aqueduct has large individual variability, which may affect the transgene efficiency.^[^ ^136^ ^]^

### 6.3 Therapeutic Window Determination

Whether there is an optimal “therapeutic time window” for gene therapy remains an interesting and important open question. At present, the age‐dependent efficacy often intermingles with other factors like delivery route.^[^ ^138^ ^]^ For example, *OTOF*‐gene therapies showed less hearing rescue in one‐month‐old mice with the posterior semicircular canal route than neonatal mice with the RWM route.^[^ ^13^, ^14^, ^26^ ^]^ However, gene therapy via the CSF route restored wild‐like ABR thresholds in two‐month‐old mice with *Slc17a8* mutations.^[^ ^32^ ^]^ In humans, *OTOF*‐gene delivery using the RWM route appears to produce the best outcome in participants aged between five and eight years old.^[^ ^23^ ^]^

On the one hand, an optimal therapeutic window may be influenced by age‐dependent neural plasticity. For example, gene therapy is applied to two groups of children with different ages. Even if the therapy corrects a peripheral defect in both groups, younger children might exhibit better outcomes than the older ones who have reduced cortical plasticity and irreversible central network damage from long‐term auditory deprivation.^[^ ^139^, ^140^ ^]^ On the other hand, the optimal window, even if it exists, may be difficult to measure in pediatric populations. This technical limitation is especially true for meaningful behavioral outcomes such as speech recognition, which depends on not only audibility but also communication and cognitive functions. Other factors like family support, linguistic environment, and socioeconomic status may also contribute to the large individual variability and the age‐dependent therapeutical window. At present, we do not understand the mechanisms underlying this optimal window, but if it exists, the clinical implication is apparent and significant.

### 6.4 Gene Editing in Genetic Hearing Loss

Here we focus on gene correction or replacement, but other gene therapies like gene editing hold great promise for broad applications. Recent advances in genome‐editing technologies (CRISPR‐Cas9 and base editors, for example) have provided expanded therapeutic strategies for both genomic and mRNA corrections.^[^ ^141^, ^142^, ^143^, ^144^, ^145^, ^146^ ^]^ However, challenges remain for gene editing in the cochlea. For example, gene off‐target risk may compromise safety, viral delivery affects cochlear cell tropism, while immune responses to bacterial Cas9 proteins are unclear.^[^ ^147^, ^148^, ^149^, ^150^ ^]^ Several novel technologies like the advanced Cas proteins^[^ ^151^, ^152^, ^153^ ^]^ or precious base/lead editing technology,^[^ ^154^ ^]^ lipid nanoparticle delivery^[^ ^155^ ^]^ and gene editing immune‐silenced or activation‐controllable Cas9 variants^[^ ^156^, ^157^, ^158^ ^]^ are being developed to address these challenges, enabling potentially new and precise therapies for deafness. Furthermore, methods such as GUIDE‐seq, CIRCLE‐seq, and DISCOVER‐seq enable genome‐wide off‐target site identification, serving as powerful tools for optimizing editing designs and assessing risks.^[^ ^159^, ^160^, ^161^ ^]^

## 7 Conclusion

As the gold standard for treating deafness, CI began humbly, facing more skepticism than support. In contrast, gene therapy for hearing loss has been launched with great promise and, if executed effectively, could transform the future of hearing healthcare. However, critical gaps must be bridged to realize this potential. Here we analyzed existing evidence and present the following six perspectives on the present status, challenges, and opportunities in gene therapy for hereditary hearing loss:

1. Gene therapy for OTOF mutations has demonstrated rapid and stable restoration of natural audibility, a feat that has never been achieved by cochlear implants or hearing aids. Existing data from 21 patients show that gene therapy, on average, improves hearing from complete loss (>100 dB HL) to moderate loss (≈50 dB HL). However, the individual variability remains large, spanning over a 90‐dB range from no effect to normal hearing.
2. Current efficacy data lack robust evidence for the restoration of critical auditory functions such as temporal processing, speech recognition in noise, sound localization, and music perception, which are essential for real‐world listening. Long‐term (two years or longer) follow‐up is needed to assess the effect of gene therapy on language development, communication skills, and hearing‐related psychosocial health. Due to the rarity of DFNB9 patients, common outcome measures beyond ABR and pure‐tone thresholds are lacking among the investigators but need to be developed to address the above complicated questions.
3. One challenge is to understand, or even predict the individual variability in outcomes through mechanistic studies of vector bio‐distribution, cellular tropism, epidemiological factors, surgical approaches, as well as baseline and follow‐up audiological and otological outcomes. Understanding the mechanisms underlying individual variability and exploring whether there is an optimal therapeutic time window for gene therapy are critical to not only advancing scientific knowledge but also meeting patients’ reasonable and realistic expectations.
4. We foresee opportunities in relaxing candidacy inclusion criteria from complete hearing loss to less loss in *OTOF* patients and expanding candidacy criteria to target other genes like *GJB2*, *STRC, SLC26A4, TMPRSS3*, which are commonly detected in the congenital hearing loss cases.
5. We expect development of alternative drug delivery methods like minimally invasive cerebrospinal fluid injection and alternative gene therapy methods like CRISPR to further improve the accessibility, safety, and efficacy in gene therapy for hearing loss.
6. Due to the well‐established evidence for quality‐of‐life improvements and cost‐effectiveness, cochlear implantation will remain the primary intervention for treating severe‐to‐profound hearing loss in the near term, whereas gene therapy will likely become an alternative to cochlear implantation in monogenic forms of deafness in the future.

## Conflict of Interest

The authors declare no conflict of interest.

## References

[1] Collaborators, G. B. D. H. L ., Lancet 2019, 397, 996.

[2] F. G. Zeng , JASA Express Lett. 2022, 2, 077201. 36154048 10.1121/10.0012825

[3] F. G. Zeng , S. Rebscher , W. Harrison , X. Sun , H. Feng , IEEE Rev. Biomed. Eng. 2008, 1, 115. 19946565 10.1109/RBME.2008.2008250 PMC2782849

[4] T. Moser , A. Starr , Nat. Rev. Neurol. 2016, 12, 135. 26891769 10.1038/nrneurol.2016.10

[5] F. G. Zeng , S. Liu , J. Speech Lang Hear. Res. 2006, 49, 367. 16671850 10.1044/1092-4388(2006/029)

[6] R. P. Carlyon , J. M. Deeks , B. Delgutte , Y. Chung , M. Vollmer , F. W. Ohl , A. Kral , J. Tillein , R. Y. Litovsky , J. Schnupp , N. Rosskothen‐Kuhl , R. L. Goldsworthy , Trends Hear. 2025, 29, 23312165251317006. 40095543 10.1177/23312165251317006 PMC12076235

[7] M. F. Dorman , S. C. Natale , J. S. Stohl , J. Felder , Front. Hum. Neurosci. 2024, 18, 1434786. 39086377 10.3389/fnhum.2024.1434786 PMC11288806

[8] F. G. Zeng , IEEE Trans. Biomed. Eng. 2017, 64, 1662. 28650803 10.1109/TBME.2017.2718939 PMC5586220

[9] R. P. Carlyon , T. Goehring , J. Assoc. Res. Otolaryngol. 2021, 22, 481. 34432222 10.1007/s10162-021-00811-5 PMC8476711

[10] A. C. Moberly , C. Bates , M. S. Harris , D. B. Pisoni , Otol. Neurotol. 2016, 37, 1522. 27631833 10.1097/MAO.0000000000001211 PMC5102802

[11] E. W. Rubel , L. A. Dew , D. W. Roberson , Science 1995, 267, 701. 7839150 10.1126/science.7839150

[12] A. G. M. Schilder , S. Wolpert , S. Saeed , L. M. Middelink , A. S. B. Edge , H. Blackshaw , A. Schilder , L. Middelink , A. Edge , A. Bibas , E. Arram , A. Bilhet , H. Cooper , E. Dalhoff , F. van Diggelen , R. J. Rutten , H. van Es , K. Hojgaard , E. Iliadou , O. Yildirim , S. Khalil , D. Kikidis , H. Lowenheim , N. Markatos , M. Mueller , T. Schade‐Mann , F. Schneider , K. Vardonikolaki , A. Wilke , K. Pastiadis , et al., Nat. Commun. 2024, 15, 1896. 38429256 10.1038/s41467-024-45784-0 PMC10907343

[13] O. Akil , F. Dyka , C. Calvet , A. Emptoz , G. Lahlou , S. Nouaille , J. Boutet de Monvel , J.‐P. Hardelin , W. W. Hauswirth , P. Avan , C. Petit , S. Safieddine , L. R. Lustig , Proc. Natl. Acad. Sci. USA 2019, 116, 4496. 30782832 10.1073/pnas.1817537116 PMC6410774

[14] H. Al‐Moyed , A. P. Cepeda , S. Jung , T. Moser , S. Kügler , E. Reisinger , EMBO Mol. Med. 2019, 11, 9396. 10.15252/emmm.201809396 PMC6328916 30509897

[15] H. Tang , H. Wang , S. Wang , S. W. Hu , J. Lv , M. Xun , K. Gao , F. Wang , Y. Chen , D. Wang , W. Wang , H. Li , Y. Shu , Hum. Genet. 2023, 142, 289. 36383253 10.1007/s00439-022-02504-2

[16] F. Zeng , Hear. J. 2024, 77, 1.

[17] L. D. Landegger , E. Reisinger , F. Lallemend , S. R. Hage , D. Grimm , C. R. Cederroth , Mol. Ther. 2025, 33, 2343. 39520052 10.1016/j.ymthe.2024.11.012 PMC12172197

[18] I. Roux , S. Safieddine , R. Nouvian , M. Grati , M.‐C. Simmler , A. Bahloul , I. Perfettini , M. Le Gall , P. Rostaing , G. Hamard , A. Triller , P. Avan , T. Moser , C. Petit , Cell 2006, 127, 277. 17055430 10.1016/j.cell.2006.08.040

[19] J. Qi , F. Tan , L. Zhang , L. Lu , S. Zhang , Y. Zhai , Y. Lu , X. Qian , W. Dong , Y. Zhou , Z. Zhang , X. Yang , L. Jiang , C. Yu , J. Liu , T. Chen , L. Wu , C. Tan , S. Sun , H. Song , Y. Shu , L. Xu , X. Gao , H. Li , R. Chai , Adv. Sci. 2024, 11, 2306788. 10.1002/advs.202306788 PMC10953563 38189623

[20] J. Qi , L. Xu , F. G. Zeng , R. Chai , Lancet 2025, 405, 777. 10.1016/S0140-6736(25)00248-X 40057333

[21] J. Lv , H. Wang , X. Cheng , Y. Chen , D. Wang , L. Zhang , Q. Cao , H. Tang , S. Hu , K. Gao , M. Xun , J. Wang , Z. Wang , B. Zhu , C. Cui , Z. Gao , L. Guo , S. Yu , L. Jiang , Y. Yin , J. Zhang , B. Chen , W. Wang , R. Chai , Z.‐Y. Chen , H. Li , Y. Shu , Lancet 2024, 403, 2317. 38280389 10.1016/S0140-6736(23)02874-X

[22] H. Wang , Y. Chen , J. Lv , X. Cheng , Q. Cao , D. Wang , L. Zhang , B. Zhu , M. Shen , C. Xu , M. Xun , Z. Wang , H. Tang , S. Hu , C. Cui , L. Jiang , Y. Yin , L. Guo , Y. Zhou , L. Han , Z. Gao , J. Zhang , S. Yu , K. Gao , J. Wang , B. Chen , W. Wang , Z.‐Y. Chen , H. Li , Y. Shu , Nat. Med. 2024, 30, 1898. 38839897 10.1038/s41591-024-03023-5 PMC11271389

[23] J. Qi , L. Zhang , L. Lu , F. Tan , C. Cheng , Y. Lu , W. Dong , Y. Zhou , X. Fu , L. Jiang , C. Tan , S. Zhang , S. Sun , H. Song , M. Duan , D. Zha , Y. Sun , X. Gao , L. Xu , F.‐G. Zeng , R. Chai , Nat. Med. 2025, 31, 2917. 40603731 10.1038/s41591-025-03773-w

[24] J. Zhang , Z. Guo , C. Pan , C. Hu , X. Weng , Y.‐W. Liu , X. Cheng , J. Lv , Q. Cao , H. Wang , Y. Chen , D. Wang , S. Hu , M. Xun , L. Zhang , Z. Wang , H. Tang , B. Zhu , L. Guo , S. Yu , X. Hu , L. Chen , B. Chen , Z.‐Y. Chen , S. Sun , X. Xu , H. Li , F. Chen , Y. Shu , Nat. Hum. Behav. 2025, 9, 1457. 40316816 10.1038/s41562-025-02184-8

[25] E. Andres‐Mateos , L. D. Landegger , C. Unzu , J. Phillips , B. M. Lin , N. A. Dewyer , J. Sanmiguel , F. Nicolaou , M. D. Valero , K. I. Bourdeu , W. F. Sewell , R. J. Beiler , M. J. McKenna , K. M. Stankovic , L. H. Vandenberghe , Nat. Commun. 2022, 13, 1359. 35292639 10.1038/s41467-022-28969-3 PMC8924271

[26] J. Qi , L. Zhang , F. Tan , Y. Zhang , Y. Zhou , Z. Zhang , H. Wang , C. Yu , L. Jiang , J. Liu , T. Chen , L. Wu , S. Zhang , S. Sun , S. Sun , L. Lu , Q. Wang , R. Chai , Adv. Sci. (Weinh) 2024, 11, 2306201. 38014592 10.1002/advs.202306201 PMC10797419

[27] L. Zhang , H. Wang , M. Xun , H. Tang , J. Wang , J. Lv , B. Zhu , Y. Chen , D. Wang , S. Hu , Z. Gao , J. Liu , Z.‐Y. Chen , B. Chen , H. Li , Y. Shu , Mol. Ther. Methods Clin. Dev. 2023, 31, 101154. 38027066 10.1016/j.omtm.2023.101154 PMC10679773

[28] Y. Chen , J. Zhong , Y. Shu , Nat. Rev. Genet. 2025, 26, 225. 39695313 10.1038/s41576-024-00809-8

[29] X. Cheng , J. Zhong , J. Zhang , C. Cui , L. Jiang , Y.‐W. Liu , Y. Chen , Q. Cao , D. Wang , G. Cheng , Y. Zong , M. Shen , C. Xu , J. Lv , H. Wang , L. Zhang , B. Zhu , H. Tang , J. Wang , X. Fan , Y. Fang , L. Guo , J. Guo , L. Chen , Y. Yin , Z. Wang , L. Han , S. Hu , S. Wang , G. Qin , et al., JAMA Neurol. 2025, 82, 941. 40690227 10.1001/jamaneurol.2025.2053 PMC12418125

[30] J. A. Greig , K. M. Martins , C. Breton , R. J. Lamontagne , Y. Zhu , Z. He , J. White , J.‐X. Zhu , J. A. Chichester , Q. Zheng , Z. Zhang , P. Bell , L. Wang , J. M. Wilson , Nat. Biotechnol. 2024, 42, 1232. 37932420 10.1038/s41587-023-01974-7 PMC11324525

[31] K. M. Martins , Hum. Gene Ther. 2023, 34, 1081. 37930949 10.1089/hum.2023.134 PMC10659022

[32] B. K. Mathiesen , L. M. Miyakoshi , C. R. Cederroth , E. Tserga , C. Versteegh , P. A. R. Bork , N. L. Hauglund , R. S. Gomolka , Y. Mori , N. K. Edvall , S. Rouse , K. Møllgård , J. R. Holt , M. Nedergaard , B. Canlon , Sci. Transl. Med. 2023, 15, abq3916. 10.1126/scitranslmed.abq3916 37379370

[33] P. T. Ranum , L. Tecedor , M. S. Keiser , Y. H. Chen , D. E. Leib , X. Liu , B. L. Davidson , Mol. Ther. 2023, 31, 609. 36610400 10.1016/j.ymthe.2022.12.018 PMC10014218

[34] D. J. Totten , K. T. A. Booth , K. M. Mosier , E. C. Cumpston , C. Whitted , V. Okechuku , N. A. Koontz , R. F. Nelson , Mol. Ther. 2023, 31, 2566. 37582360 10.1016/j.ymthe.2023.08.001 PMC10492018

[35] F. G. Zeng , S. Oba , S. Garde , Y. Sininger , A. Starr , Neuroreport 1999, 10, 3429. 10599857 10.1097/00001756-199911080-00031

[36] J. Bourien , Y. Tang , C. Batrel , A. Huet , M. Lenoir , S. Ladrech , G. Desmadryl , R. Nouvian , J.‐L. Puel , J. Wang , J. Neurophysiol. 2014, 112, 1025. 24848461 10.1152/jn.00738.2013

[37] Y. Yuan , F. Shi , Y. Yin , M. Tong , H. Lang , D. B. Polley , M. C. Liberman , A. S. B. Edge , J. Assoc. Res. Otolaryngol. 2014, 15, 31. 24113829 10.1007/s10162-013-0419-7 PMC3901858

[38] L. Zhang , Med 2025, 6, 100696. 40409265 10.1016/j.medj.2025.100696

[39] A. Starr , T. W. Picton , Y. Sininger , L. J. Hood , C. I. A. Berlin , Brain 1996, 119, 741. 8673487 10.1093/brain/119.3.741

[40] F. G. Zeng , Y. Y. Kong , H. J. Michalewski , A. Starr , J. Neurophysiol. 2005, 93, 3050. 15615831 10.1152/jn.00985.2004

[41] R. V. Shannon , F. G. Zeng , V. Kamath , J. Wygonski , M. Ekelid , Science 1995, 270, 303. 7569981 10.1126/science.270.5234.303

[42] M. C. Killion , P. A. Niquette , G. I. Gudmundsen , L. J. Revit , S. Banerjee , J. Acoust. Soc. Am. 2004, 116, 2395. 15532670 10.1121/1.1784440

[43] M. F. Dorman , L. H. Loiselle , S. J. Cook , W. A. Yost , R. H. Gifford , Audiol. Neuro‐Otol. 2016, 21, 127. 10.1159/000444740 PMC4949120 27077663

[44] A. F. Mahmoud , M. J. Ruckenstein , Otol. Neurotol. 2014, 35, 286. 10.1097/MAO.0000000000000581 25226375

[45] J. A. Beyea , K. P. McMullen , M. S. Harris , D. M. Houston , J. M. Martin , V. A. Bolster , O. F. Adunka , A. C. Moberly , Otol. Neurotol. 2016, 37, 1238. 27466894 10.1097/MAO.0000000000001162

[46] A. A. Eshraghi , J. Ahmed , E. Krysiak , K. Ila , P. Ashman , F. F. Telischi , S. Angeli , S. Prentiss , D. Martinez , S. Valendia , Acta Otolaryngol. 2017, 137, 384. 27918225 10.1080/00016489.2016.1256499

[47] B. G. Kim , JAMA Otolaryngol. Head Neck Surg. 2013, 139, 604. 23787419 10.1001/jamaoto.2013.3195

[48] C. Olds , L. Pollonini , H. Abaya , J. Larky , M. Loy , H. Bortfeld , M. S. Beauchamp , J. S. Oghalai , Ear Hear. 2016, 37, 160. 10.1097/AUD.0000000000000258 PMC4844823 26709749

[49] S. F. M. Hashemi , M. Rajati , R. Yousefi , M. M. Ghasemi , H. Tayarani , M. R. Tale , Eur. Arch. Otorhinolaryngol. 2023, 280, 5319. 37378728 10.1007/s00405-023-08054-1

[50] F. G. Zeng , R. V. Shannon , Neuroreport 1999, 10, 1931. 10501535 10.1097/00001756-199906230-00025

[51] R. V. Shannon , J. Acoust. Soc. Am. 1992, 91, 2156. 1597606 10.1121/1.403807

[52] A. J. Oxenham , J. Neurosci. 2012, 32, 13335. 23015422 10.1523/JNEUROSCI.3815-12.2012 PMC3481156

[53] F.‐G. Zeng , K. Nie , G. S. Stickney , Y.‐Y. Kong , M. Vongphoe , A. Bhargave , C. Wei , K. Cao , Proc. Natl. Acad. Sci. USA 2005, 102, 2293. 15677723 10.1073/pnas.0406460102 PMC546014

[54] Y. Kong , G. Stickney , F. Zeng , J. Acoust. Soc. Am. 2005, 117, 1351. 15807023 10.1121/1.1857526

[55] C. A. von Ilberg , U. Baumann , J. Kiefer , J. Tillein , O. F. Adunka , Audiol. Neurootol. 2011, 16, 1. 10.1159/000327765 21606646

[56] A. K. Cheng , JAMA, J. Am. Med. Assoc. 2000, 284, 850.

[57] I. Cejas , D. H. Barker , E. Petruzzello , C. M. Sarangoulis , A. L. Quittner , JAMA Otolaryngol. Head Neck Surg. 2023, 149, 708. 37382935 10.1001/jamaoto.2023.1327 PMC10311426

[58] S. Rebscher , D. D. Zhou , F. G. Zeng , J. Int. Adv. Otol. 2018, 14, 392. 30644380 10.5152/iao.2018.6285 PMC6354516

[59] C. H. Wong , D. Li , N. Wang , J. Gruber , A. W. Lo , R. M. Conti , Gene Ther. 2023, 30, 761. 37935855 10.1038/s41434-023-00419-9 PMC10678302

[60] D. Duman , A. Sirmaci , F. B. Cengiz , H. Ozdag , M. Tekin , Genet. Test Mol. Biomarkers 2011, 15, 29. 21117948 10.1089/gtmb.2010.0120

[61] C. C. Wu , Genes 2019, 10, 338.31064091

[62] N. Mahdieh , A. Shirkavand , B. Rabbani , M. Tekin , B. Akbari , M. T. Akbari , S. Zeinali , Int. J. Pediatr. Otorhinolaryngol. 2012, 76, 1610. 22906306 10.1016/j.ijporl.2012.07.030

[63] C. M. Sloan‐Heggen , A. O. Bierer , A. E. Shearer , D. L. Kolbe , C. J. Nishimura , K. L. Frees , S. S. Ephraim , S. B. Shibata , K. T. Booth , C. A. Campbell , P. T. Ranum , A. E. Weaver , E. A. Black‐Ziegelbein , D. Wang , H. Azaiez , R. J. H. Smith , Hum. Genet. 2016, 135, 441. 26969326 10.1007/s00439-016-1648-8 PMC4796320

[64] A. Tropitzsch , Ear Hear. 2022, 43, 1049. 34753855 10.1097/AUD.0000000000001159 PMC9007094

[65] B. Choi , Z. Ahmed , S. Riazuddin , M. Bhinder , M. Shahzad , T. Husnain , S. Riazuddin , A. Griffith , T. Friedman , Clin. Genet. 2009, 75, 237. 19250381 10.1111/j.1399-0004.2008.01128.x PMC3461579

[66] E. M. Richard , R. L. P. Santos‐Cortez , R. Faridi , A. U. Rehman , K. Lee , M. Shahzad , A. Acharya , A. A. Khan , A. Imtiaz , I. Chakchouk , C. Takla , I. Abbe , M. Rafeeq , K. Liaqat , T. Chaudhry , M. J. Bamshad , D. A. Nickerson , I. Schrauwen , S. N. Khan , R. J. Morell , S. Zafar , M. Ansar , Z. M. Ahmed , W. Ahmad , S. Riazuddin , T. B. Friedman , S. M. Leal , S. Riazuddin , Hum. Mutat. 2019, 40, 53. 30303587 10.1002/humu.23666 PMC6296877

[67] D. Baux , C. Vaché , C. Blanchet , M. Willems , C. Baudoin , M. Moclyn , V. Faugère , R. Touraine , B. Isidor , D. Dupin‐Deguine , M. Nizon , M. Vincent , S. Mercier , C. Calais , G. García‐García , Z. Azher , L. Lambert , Y. Perdomo‐Trujillo , F. Giuliano , M. Claustres , M. Koenig , M. Mondain , A. F. Roux , Sci. Rep. 2017, 7, 16783. 29196752 10.1038/s41598-017-16846-9 PMC5711943

[68] Y.‐I. Iwasa , S.‐Y. Nishio , A. Sugaya , Y. Kataoka , Y. Kanda , M. Taniguchi , K. Nagai , Y. Naito , T. Ikezono , R. Horie , Y. Sakurai , R. Matsuoka , H. Takeda , S. Abe , C. Kihara , T. Ishino , S.‐Y. Morita , S. Iwasaki , M. Takahashi , T. Ito , Y. Arai , S.‐I. Usami , PLoS One 2019, 14, 0215932. 10.1371/journal.pone.0215932 PMC6522017 31095577

[69] J. Wu , Z. Cao , Y. Su , Y. Wang , R. Cai , J. Chen , B. Gao , M. Han , X. Li , D. Zhang , X. Gao , S. Huang , Q. Huang , Y. Yuan , X. Ma , P. Dai , J. Hum. Genet. 2022, 67, 643. 35982127 10.1038/s10038-022-01066-5 PMC9592555

[70] L. Zhang , F. Tan , J. Qi , Y. Lu , X. Wang , X. Yang , X. Chen , X. Zhang , J. Fan , Y. Zhou , L. Peng , N. Li , L. Xu , S. Yang , R. Chai , Adv. Sci. 2024, 11, 2402166. 10.1002/advs.202402166 PMC11653604 39556694

[71] D. K. Chan , K. W. Chang , Laryngoscope 2014, 124, E34. 23900770 10.1002/lary.24332

[72] A. Tlili , M. Mahfood , A. A. Mutery , J. Chouchen , Hum. Genom. 2024, 18, 59. 10.1186/s40246-024-00630-8 PMC11157727 38844983

[73] I. D. Castillo , M. M , D.‐R. M , M.‐P. MA , Hum. Genet. 2022, 141, 683.35044523

[74] C.‐S. M , T. Ott , V. Michel , J. P. Hardelin , I. Perfettini , M. Eybalin , T. Wu , D. C. Marcus , P. Wangemann , K. Willecke , C. Petit , Curr. Biol. 2022, 12, 1106. 10.1016/s0960-9822(02)00904-1 PMC4030438 12121617

[75] L. Mei , Neurobiol. Dis. 2017, 108, 195. 28823936 10.1016/j.nbd.2017.08.002 PMC5675824

[76] S. Y. Nishio , S. I. Usami , Sci. Rep. 2022, 12, 634. 35022556 10.1038/s41598-021-04688-5 PMC8755823

[77] V. E , M. Leibovici , N. Michalski , R. J. Goodyear , C. Houdon , D. Weil , G. P. Richardson , C. Petit , J. Compar. Neurol. 2011, 519, 194. 10.1002/cne.22509 PMC3375590 21165971

[78] H. J. Park , J. Med. Genet. 2003, 40, 242. 12676893 10.1136/jmg.40.4.242 PMC1735432

[79] T. Yoshino , et al., Hear. Res. 2004, 195, 9. 15350275 10.1016/j.heares.2004.05.005

[80] I. A. Belyantseva , E. T. Boger , T. B. Friedman , Proc. Natl. Acad. Sci. USA 2003, 100, 13958. 14610277 10.1073/pnas.2334417100 PMC283528

[81] G. Aparna , PLoS One 2014, 9, 84773.

[82] L. Jinwook , Gene 2013, 532, 276.23958653

[83] C. Yuan‐Siao , J. Med. Genet. 2022, 59, 1219.35961784

[84] L. Wei , Cell Tissue Res. 2018, 372, 445. 29460002 10.1007/s00441-018-2793-2 PMC5949142

[85] L. Sihan , Nat. Commun. 2020, 11, 2066. 32350269 10.1038/s41467-020-15936-z PMC7190839

[86] S. Yasunaga , Nat. Genet. 1999, 21, 363. 10192385 10.1038/7693

[87] P. K. Legan , A. Rau , J. N. Keen , G. P. Richardson , J. Biol. Chem. 1997, 272, 8791. 9079715 10.1074/jbc.272.13.8791

[88] J. Yang , PLoS Genet. 2010, 6, 1000955.

[89] M. Miyagawa , S. Y. Nishio , S.‐I. Usami , PLoS One 2012, 7, 40366. 10.1371/journal.pone.0040366 PMC3416829 22899989

[90] A. Lagziel , Dev. Biol. 2005, 280, 295. 15882574 10.1016/j.ydbio.2005.01.015

[91] T. Kharkovets , Proc. Natl. Acad. Sci. USA 2000, 97, 4333. 10760300 10.1073/pnas.97.8.4333 PMC18242

[92] K. Kiyoto , Cell Rep. 2015, 12, 1606.26321635

[93] W. Wei‐Qian , Front. Genet. 2022, 13, 825082. 35711932 10.3389/fgene.2022.825082 PMC9196635

[94] M. Karuna , Genes 2019, 10, 735.31547530

[95] G. Nicolas , Am. J. Hum. Genet. 2009, 85, 328.19732867

[96] T. Alix , J. Neurosci. 2021, 41, 3331.33707295

[97] M. Eiji , et al., Exp. Anim. 2010, 59, 57.20224170

[98] S. Yuta , PLoS One 2017, 12, 0183477.

[99] M. Vincent , Sci. Rep. 2020, 10, 16430.33009420

[100] R. J. Carlson , T. Walsh , J. B. Mandell , A. Aburayyan , M. K. Lee , S. Gulsuner , D. L. Horn , H. C. Ou , K. C. Y. Sie , L. Mancl , J. Rubinstein , M.‐C. King , JAMA Otolaryngol. Head Neck Surg. 2023, 149, 212. 36633841 10.1001/jamaoto.2022.4463 PMC9857764

[101] F. Denoyelle , G. Lina‐Granade , H. Plauchu , R. Bruzzone , H. Chaïb , F. Lévi‐Acobas , D. Weil , C. Petit , Nature 1998, 393, 319. 9620796 10.1038/30639

[102] D. P. Kelsell , J. Dunlop , H. P. Stevens , N. J. Lench , J. N. Liang , G. Parry , R. F. Mueller , I. M. Leigh , Nature 1997, 387, 80. 9139825 10.1038/387080a0

[103] L. Zelante , Hum. Mol. Genet. 1997, 6, 1605. 9285800 10.1093/hmg/6.9.1605

[104] F. Denoyelle , D. Weil , M. A. Maw , S. A. Wilcox , N. J. Lench , D. R. Allen‐Powell , A. H. Osborn , H.‐H. M. Dahl , A. Middleton , M. J. Houseman , C. Dode , S. Marlin , A. Boulila‐ElGaied , M. Grati , H. Ayadi , S. BenArab , P. Bitoun , G. Lina‐Granade , J. Godet , M. Mustapha , J. Loiselet , E. El‐Zir , A. Aubois , A. Joannard , J. Levilliers , E.‐N. Garabedian , R. F. Mueller , R. J. McKinlay Gardner , C. Petit , Hum. Mol. Genet. 1997, 6, 2173. 9336442 10.1093/hmg/6.12.2173

[105] Y. P. Liu , H. B. Zhao , Cell Tissue Res. 2008, 333, 395. 18581144 10.1007/s00441-008-0641-5 PMC2548271

[106] J. Guo , X. Ma , J. M. Skidmore , J. Cimerman , D. M. Prieskorn , L. A. Beyer , D. L. Swiderski , D. F. Dolan , D. M. Martin , Y. Raphael , Mol. Ther. Methods Clin. Dev. 2021, 23, 319. 34729379 10.1016/j.omtm.2021.09.009 PMC8531464

[107] F. Denoyelle , S. Marlin , D. Weil , L. Moatti , P. Chauvin , É.‐N. Garabédian , C. Petit , Lancet 1999, 353, 1298. 10218527 10.1016/S0140-6736(98)11071-1

[108] H.‐D. Gabriel , D. Jung , C. Bützler , A. Temme , O. Traub , E. Winterhager , K. Willecke , J. Cell Biol. 1998, 140, 1453. 9508777 10.1083/jcb.140.6.1453 PMC2132681

[109] Q. Sun , F. Tan , L. Zhang , Y. Lu , H. Wei , N. Li , L. Jiang , Y. Zhou , T. Chen , L. Lu , G.‐L. Li , J. Qi , S. Yang , R. Chai , Mol. Ther. 2025, 33, 3006. 40121530 10.1016/j.ymthe.2025.03.029 PMC12266026

[110] Q. Li , C. Cui , R. Liao , X. Yin , D. Wang , Y. Cheng , B. Huang , L. Wang , M. Yan , J. Zhou , J. Zhao , W. Tang , Y. Wang , X. Wang , J. Lv , J. Li , H. Li , Y. Shu , Cell. Mol. Life Sci. 2023, 80, 148. 37178259 10.1007/s00018-023-04794-9 PMC10182940

[111] R. J. Carlson , S. Taiber , J. T. Rubinstein , Otol. Neurotol. 2025, 46, 239. 39951658 10.1097/MAO.0000000000004423

[112] W. Du , V. Ergin , C. Loeb , M. Huang , S. Silver , A. M. Armstrong , Z. Huang , C. B. Gurumurthy , H. Staecker , X. Liu , Z.‐Y. Chen , Mol. Ther. 2023, 31, 2796. 37244253 10.1016/j.ymthe.2023.05.005 PMC10491991

[113] M. V. Ivanchenko , D. M. Hathaway , A. J. Klein , B. Pan , O. Strelkova , P. De‐la‐Torre , X. Wu , C. W. Peters , E. M. Mulhall , K. T. Booth , C. Goldstein , J. Brower , M. Sotomayor , A. A. Indzhykulian , D. P. Corey , Nat. Commun. 2023, 14, 2400. 37100771 10.1038/s41467-023-38038-y PMC10133396

[114] C. Askew , C. Rochat , B. Pan , Y. Asai , H. Ahmed , E. Child , B. L. Schneider , P. Aebischer , J. R. Holt , Sci. Transl. Med. 2015, 7, 295ra108. 10.1126/scitranslmed.aab1996 PMC7298700 26157030

[115] C. A. Nist‐Lund , Nat. Commun. 2019, 10, 236. 30670701 10.1038/s41467-018-08264-w PMC6342993

[116] J. Wu , P. Solanes , C. Nist‐Lund , S. Spataro , O. Shubina‐Oleinik , I. Marcovich , H. Goldberg , B. L. Schneider , J. R. Holt , Mol. Ther. 2021, 29, 973. 33212302 10.1016/j.ymthe.2020.11.016 PMC7934577

[117] X. Zhao , H. Liu , H. Liu , R. Cai , H. Wu , Hum. Gene Ther. 2022, 33, 729. 35726398 10.1089/hum.2022.062

[118] O. Shubina‐Oleinik , C. Nist‐Lund , C. French , S. Rockowitz , A. E. Shearer , J. R. Holt , Sci. Adv. 2021, 7, abi7629. 10.1126/sciadv.abi7629 PMC8673757 34910522

[119] S. Hadi , A. J. Alexander , A. C. Velez‐Ortega , G. I. Frolenkov , J. Assoc. Res. Otolaryngol. 2020, 21, 121. 32152769 10.1007/s10162-020-00745-4 PMC7271090

[120] R. H. Holme , K. P. Steel , Hear. Res. 2002, 169, 13. 12121736 10.1016/s0378-5955(02)00334-9

[121] T. Hutchin , N. Coy , H. Conlon , E. Telford , K. Bromelow , D. Blaydon , G. Taylor , E. Coghill , S. Brown , R. Trembath , X. Liu , M. Bitner‐Glindzicz , R. Mueller , Clin. Genet. 2005, 68, 506. 16283880 10.1111/j.1399-0004.2005.00539.x

[122] M.‐A. Kim , S. H. Kim , N. Ryu , J.‐H. Ma , Y.‐R. Kim , J. Jung , C.‐J. Hsu , J. Y. Choi , K.‐Y. Lee , P. Wangemann , J. Bok , U.‐K. Kim , Theranostics 2019, 9, 7184. 31695761 10.7150/thno.38032 PMC6831294

[123] Z. Wu , H. Yang , P. Colosi , Mol. Ther. 2010, 18, 80. 19904234 10.1038/mt.2009.255 PMC2839202

[124] H. Yoshimura , S. Yokota , Y. Takumi , Curr. Issues Mol. Biol. 2023, 45, 9413. 38132436 10.3390/cimb45120590 PMC10741579

[125] Q. Ling , J. A. Herstine , A. Bradbury , S. J. Gray , Nat. Rev. Drug Discovery 2023, 22, 789. 37658167 10.1038/s41573-023-00766-7

[126] M. Abbas , J. Wang , N. Leboucq , M. Mondain , F. Blanc , J. Assoc. Res. Otolaryngol. 2024, 25, 611. 39294515 10.1007/s10162-024-00963-0 PMC11683029

[127] H. Yi , Am. J. Transl. Res. 2016, 8, 5494. 28078020 PMC5209500

[128] A. F. Ghiz , A. N. Salt , J. E. DeMott , M. M. Henson , O. W. Henson , S. L. Gewalt , Hear. Res. 2001, 162, 105. 11707357 10.1016/s0378-5955(01)00375-6

[129] R. Manrique‐Huarte , M. A. D. Linera‐Alperi , D. Parilli , J. Rodriguez , D. Borro , W. Dueck , D. Smyth , A. Salt , M. Manrique , Hear. Res. 2021, 404, 108228. 33784550 10.1016/j.heares.2021.108228

[130] H. Costa‐Verdera , V. Meneghini , Z. Fitzpatrick , M. Abou Alezz , E. Fabyanic , X. Huang , Y. Dzhashiashvili , A. Ahiya , E. Mangiameli , E. Valeri , G. Crivicich , S. Piccolo , I. Cuccovillo , R. Caccia , Y. K. Chan , B. Bertin , G. Ronzitti , E. A. Engel , I. Merelli , F. Mingozzi , A. Gritti , K. Kuranda , A. Kajaste‐Rudnitski , Nat. Commun. 2025, 16, 3694. 40251179 10.1038/s41467-025-58778-3 PMC12008376

[131] C. Hinderer , N. Katz , E. L. Buza , C. Dyer , T. Goode , P. Bell , L. K. Richman , J. M. Wilson , Hum. Gene Ther. 2018, 29, 285. 29378426 10.1089/hum.2018.015 PMC5865262

[132] J. Hordeaux , E. L. Buza , C. Dyer , T. Goode , T. W. Mitchell , L. Richman , N. Denton , C. Hinderer , N. Katz , R. Schmid , R. Miller , G. R. Choudhury , M. Horiuchi , K. Nambiar , H. Yan , M. Li , J. M. Wilson , Hum. Gene Ther. 2020, 31, 808. 32845779 10.1089/hum.2020.167

[133] Z. C. E. Hawley , I. D. Pardo , S. Cao , M. I. Zavodszky , F. Casey , K. Ferber , Y. Luo , S. Hana , S. K. Chen , J. Doherty , R. Costa , P. Cullen , Y. Liu , T. M. Carlile , T. Chowdhury , B. Doyle , P. Clarner , K. Mangaudis , E. Guilmette , S. Bourque , D. Koske , M. V. P. Nadella , P. Trapa , M. L. Hawes , D. Raitcheva , S.‐C. Lo , Mol. Ther. 2025, 33, 215. 39563026 10.1016/j.ymthe.2024.11.029 PMC11764093

[134] O. Akil , R. P. Seal , K. Burke , C. Wang , A. Alemi , M. During , R. H. Edwards , L. R. Lustig , Neuron 2012, 75, 283. 22841313 10.1016/j.neuron.2012.05.019 PMC3408581

[135] M. Neiger , Fortschr. Hals Nasen Ohrenheilkd 1968, 15, 113. 4978651

[136] Q. Gopen , J. J. Rosowski , S. N. Merchant , Hear. Res. 1997, 107, 9. 9165342 10.1016/s0378-5955(97)00017-8

[137] J. A. Burton , M. D. Valero , T. A. Hackett , R. Ramachandran , J. Acoust. Soc. Am. 2019, 146, 3770. 31795680 10.1121/1.5132709 PMC6881191

[138] S. Iranfar , M. Cornille , M. S. Roldan , B. Plion , M.‐J. Lecomte , S. Safieddine , G. Lahlou , Sci. Rep. 2025, 15, 13479. 40251388 10.1038/s41598-025-98007-x PMC12008179

[139] A. Sharma , M. F. Dorman , A. J. Spahr , Ear. Hear. 2002, 23, 532. 12476090 10.1097/00003446-200212000-00004

[140] M. Stropahl , S. Debener , Neuroimage Clin. 2017, 16, 514. 28971005 10.1016/j.nicl.2017.09.001 PMC5609862

[141] Y. Xue , Y. Tao , X. Wang , X. Wang , Y. Shu , Y. Liu , W. Kang , S. Chen , Z. Cheng , B. Yan , Y. Xie , L. Bi , H. Jia , J. Li , Q. Xiao , L. Chen , X. Yao , L. Shi , H. Yang , H. Wu , Mol. Ther. 2023, 31, 3520. 37915172 10.1016/j.ymthe.2023.10.019 PMC10727966

[142] C. Cui , S. Wang , D. Wang , J. Zhao , B. Huang , B. Zhu , Y. Chen , H. Tang , Y. Han , C. Ye , D. Mu , C. Zhang , Y. Yang , Y. Bao , J. Lv , S. Han , G.‐L. Li , H. Li , Y. Shu , Nat. Biomed. Eng. 2025, 9, 40. 39134683 10.1038/s41551-024-01235-1

[143] W.‐H. Yeh , O. Shubina‐Oleinik , J. M. Levy , B. Pan , G. A. Newby , M. Wornow , R. Burt , J. C. Chen , J. R. Holt , D. R. Liu , Sci. Transl. Med. 2020, 12, aay9101. 10.1126/scitranslmed.aay9101 PMC8167884 32493795

[144] Q. Xiao , Z. Xu , Y. Xue , C. Xu , L. Han , Y. Liu , F. Wang , R. Zhang , S. Han , X. Wang , G.‐L. Li , H. Li , H. Yang , Y. Shu , Sci. Transl. Med. 2022, 14, abn0449. 10.1126/scitranslmed.abn0449 35857824

[145] Z. Zheng , G. Li , C. Cui , F. Wang , X. Wang , Z. Xu , H. Guo , Y. Chen , H. Tang , D. Wang , M. Huang , Z.‐Y. Chen , X. Huang , H. Li , G.‐L. Li , X. Hu , Y. Shu , Signal Transduction Targeted Ther. 2022, 7, 79. 10.1038/s41392-022-00893-4 PMC8918553 35283480

[146] Y. Xue , X. Hu , D. Wang , D. Li , Y. Li , F. Wang , M. Huang , X. Gu , Z. Xu , J. Zhou , J. Wang , R. Chai , J. Shen , Z.‐Y. Chen , G.‐L. Li , H. Yang , H. Li , E. Zuo , Y. Shu , Mol. Ther. 2022, 30, 105. 34174443 10.1016/j.ymthe.2021.06.015 PMC8753286

[147] A. M. Keeler , W. Zhan , S. Ram , K. A. Fitzgerald , G. Gao , Mol. Ther. 2025, 33, 1946. 40156190 10.1016/j.ymthe.2025.03.037 PMC12126790

[148] C. T. Charlesworth , P. S. Deshpande , D. P. Dever , J. Camarena , V. T. Lemgart , M. K. Cromer , C. A. Vakulskas , M. A. Collingwood , L. Zhang , N. M. Bode , M. A. Behlke , B. Dejene , B. Cieniewicz , R. Romano , B. J. Lesch , N. Gomez‐Ospina , S. Mantri , M. Pavel‐Dinu , K. I. Weinberg , M. H. Porteus , Nat. Med. 2019, 25, 249. 30692695 10.1038/s41591-018-0326-x PMC7199589

[149] D. L. Wagner , L. Amini , D. J. Wendering , L.‐M. Burkhardt , L. Akyüz , P. Reinke , H.‐D. Volk , M. Schmueck‐Henneresse , Nat. Med. 2019, 25, 242. 30374197 10.1038/s41591-018-0204-6

[150] W. L. Chew , M. Tabebordbar , J. K. W. Cheng , P. Mali , E. Y. Wu , A. H. M. Ng , K. Zhu , A. J. Wagers , G. M. Church , Nat. Methods 2016, 13, 868. 27595405 10.1038/nmeth.3993 PMC5374744

[151] B. P. Kleinstiver , V. Pattanayak , M. S. Prew , S. Q. Tsai , N. T. Nguyen , Z. Zheng , J. K. Joung , Nature 2016, 529, 490. 26735016 10.1038/nature16526 PMC4851738

[152] J. S. Chen , Y. S. Dagdas , B. P. Kleinstiver , M. M. Welch , A. A. Sousa , L. B. Harrington , S. H. Sternberg , J. K. Joung , A. Yildiz , J. A. Doudna , Nature 2017, 550, 407. 28931002 10.1038/nature24268 PMC5918688

[153] I. M. Slaymaker , L. Gao , B. Zetsche , D. A. Scott , W. X. Yan , F. Zhang , Science 2016, 351, 84. 26628643 10.1126/science.aad5227 PMC4714946

[154] J. A. Zuris , D. B. Thompson , Y. Shu , J. P. Guilinger , J. L. Bessen , J. H. Hu , M. L. Maeder , J. K. Joung , Z.‐Y. Chen , D. R. Liu , Nat. Biotechnol. 2015, 33, 73. 25357182 10.1038/nbt.3081 PMC4289409

[155] X. Gao , Y. Tao , V. Lamas , M. Huang , W.‐H. Yeh , B. Pan , Y.‐J. Hu , J. H. Hu , D. B. Thompson , Y. Shu , Y. Li , H. Wang , S. Yang , Q. Xu , D. B. Polley , M. C. Liberman , W.‐J. Kong , J. R. Holt , Z.‐Y. Chen , D. R. Liu , Nature 2018, 553, 217. 29258297 10.1038/nature25164 PMC5784267

[156] J. Lu , C. Zhao , Y. Zhao , J. Zhang , Y. Zhang , L. Chen , Q. Han , Y. Ying , S. Peng , R. Ai , Y. Wang , Nucleic Acids Res. 2018, 46, 25. 29237052 10.1093/nar/gkx1222 PMC5861443

[157] S. R. Ferdosi , R. Ewaisha , F. Moghadam , S. Krishna , J. G. Park , M. R. Ebrahimkhani , S. Kiani , K. S. Anderson , Nat. Commun. 2019, 10, 1842. 31015529 10.1038/s41467-019-09693-x PMC6478683

[158] R. Raghavan , M. J. Friedrich , I. King , S. Chau‐Duy‐Tam Vo , D. Strebinger , B. Lash , M. Kilian , M. Platten , R. K. Macrae , Y. Song , L. Nivon , F. Zhang , Nat. Commun. 2025, 16, 105. 39747875 10.1038/s41467-024-55522-1 PMC11696374

[159] S. Q. Tsai , Z. Zheng , N. T. Nguyen , M. Liebers , V. V. Topkar , V. Thapar , N. Wyvekens , C. Khayter , A. J. Iafrate , L. P. Le , M. J. Aryee , J. K. Joung , Nat. Biotechnol. 2015, 33, 187. 25513782 10.1038/nbt.3117 PMC4320685

[160] S. Q. Tsai , N. T. Nguyen , J. Malagon‐Lopez , V. V. Topkar , M. J. Aryee , J. K. Joung , Nat. Methods 2017, 14, 607. 28459458 10.1038/nmeth.4278 PMC5924695

[161] B. Wienert , S. K. Wyman , C. D. Richardson , C. D. Yeh , P. Akcakaya , M. J. Porritt , M. Morlock , J. T. Vu , K. R. Kazane , H. L. Watry , L. M. Judge , B. R. Conklin , M. Maresca , J. E. Corn , Science 2019, 364, 286. 31000663 10.1126/science.aav9023 PMC6589096

